# Human Neural Stem Cells Overexpressing Choline Acetyltransferase Restore Unconditioned Fear in Rats with Amygdala Injury

**DOI:** 10.1155/2016/8521297

**Published:** 2016-03-21

**Authors:** Kyungha Shin, Yeseul Cha, Kwang Sei Kim, Ehn-Kyoung Choi, Youngjin Choi, Haiyu Guo, Young-Hwan Ban, Jong-Choon Kim, Dongsun Park, Yun-Bae Kim

**Affiliations:** ^1^College of Veterinary Medicine, Chungbuk National University, Cheongju 28644, Republic of Korea; ^2^College of Veterinary Medicine, Chonnam National University, Gwangju 61186, Republic of Korea; ^3^Department of Physiology, Ajou University School of Medicine, Suwon 16499, Republic of Korea

## Abstract

Amygdala is involved in the fear memory that recognizes certain environmental cues predicting threatening events. Manipulation of neurotransmission within the amygdala affects the expression of conditioned and unconditioned emotional memories such as fear freezing behaviour. We previously demonstrated that F3.ChAT human neural stem cells (NSCs) overexpressing choline acetyltransferase (ChAT) improve cognitive function of Alzheimer's disease model rats with hippocampal or cholinergic nerve injuries by increasing acetylcholine (ACh) level. In the present study, we examined the effect of F3.ChAT cells on the deficit of unconditioned fear freezing. Rats given N-methyl-d-aspartate (NMDA) in their amygdala 2 weeks prior to cat odor exposure displayed very short resting (freezing) time compared to normal animals. NMDA induced neuronal degeneration in the amygdala, leading to a decreased ACh concentration in cerebrospinal fluid. However, intracerebroventricular transplantation of F3.ChAT cells attenuated amygdala lesions 4 weeks after transplantation. The transplanted cells were found in the NMDA-injury sites and produced ChAT protein. In addition, F3.ChAT-receiving rats recuperated freezing time staying remote from the cat odor source, according to the recovery of brain ACh concentration. The results indicate that human NSCs overexpressing ChAT may facilitate retrieval of unconditioned fear memory by increasing ACh level.

## 1. Introduction

It is well known that acquisition and processing (consolidation) of habituation memory mainly occur in the hippocampus. Thus, destruction of the hippocampus with toxic chemicals such as kainic acid causes cognitive deficit [1, 2]. By comparison, amygdala governs emotional memory such as fear conditioning [3]. Notably, each part of amygdala is involved in the fear processing and/or expression of fear-related behaviours; basolateral nucleus plays a role in the contextual fear conditioning, while medial nucleus elicits the activation of unconditioned fear [4–6]. Unconditioned fear, believed to be innate or instinctive, is explained as a cluster of behavioural phenomena when an animal faces a threatening event [7]. For instance, wild and laboratory rats instinctively show fear responses, that is, freezing, retreating, or hiding, to the odor of their predators such as cats, foxes, and ferrets, even though they have no former experience facing the predators.

Destruction or inactivation of medial amygdala led to a decrease in the fear freezing response to predators' odors including trimethylthiazoline, a component of fox feces [3, 4, 6]. Therefore, it is expected that the amygdala is one of the key regions for insight into the unconditioned fear (also called instinct), although it is unlikely that the elucidation on the nature of instinct would be achieved in the near future. Whether being of genetic nature or constructed during neural system development, it is believed that the unconditioned fear is a kind of memory rather than a reflex seen in avoidance from a burning pain. Recently, it was demonstrated that humans with bilateral amygdala damage felt fear and panic following 35% CO_2_ inhalation [8], implying that the amygdala may not be the storage site of emotional (fear) memory. Thus, it is expected that protection or restoration of neurotransmission in the amygdala may recover the unconditioned fear memory. Notably, it was reported that muscarinic receptors in the amygdala and infralimbic neurons govern trace fear conditioning and fear extinction [9, 10], indicative of an important role of acetylcholine (ACh) released from cholinergic nervous system.

Regenerative medicine using stem cells has been emerged as a promising strategy to treat neurodegenerative diseases including Alzheimer's disease (AD) [11, 12]. Recently, we established a human neural stem cell line (F3.ChAT) encoding gene of choline acetyltransferase (ChAT), an enzyme responsible for ACh synthesis. In our previous studies, it was demonstrated that transplantation of F3.ChAT stem cells overexpressing ChAT mRNA restored cognitive function in AD model rats with hippocampus or cholinergic nerve injuries as well as in aging animals by increasing brain ACh level [1, 13, 14]. In addition to the effects on conditioned fear acquisition from electric shock and water swimming during passive avoidance and water-maze trials in AD and aging models, we investigated the effects of F3.ChAT cell transplantation on the retrieval of unconditioned fear memory for cat odor in amygdala-destroyed rats.

## 2. Methods

### 2.1. Human F3.ChAT Cells and Immunocytochemistry

An immortalized NSC line, HB1.F3 (F3), was established from primary cultures of a 15-week gestational human fetal brain by infecting primary cultured NSC with a retroviral vector encoding v-myc oncogene [15, 16]. F3.ChAT cells were prepared by infecting a human F3 NSC line with a retroviral vector encoding human ChAT gene as described previously [1, 13, 14]. F3.ChAT cells were plated on poly-l-lysine-coated Aclar plastic coverslips and fixed with 4% paraformaldehyde in 0.1 M phosphate buffer for 5 min at room temperature (RT). Fixed cultures were incubated with primary antibody specific for human nestin (1 : 500, mouse monoclonal, Chemicon, Temecula, CA, USA), or human ChAT (1 : 200, mouse monoclonal, Chemicon) for 24 hr at 4°C, followed by Alexa Fluor 488-conjugated anti-mouse IgG or Alexa Fluor 594-conjugated anti-rabbit IgG (1 : 1,000; Molecular Probes, Eugene, OR, USA) for 1 hr at RT. Cells were observed under an Olympus laser-scanning confocal microscope (LSM710; Zeiss, New York, NY, USA).

### 2.2. Amygdala Injury Animal Model and Cell Transplantation

Male Sprague-Dawley rats (*n* = 8/group, Orient-Bio, Seongnam, Korea) weighing within 220–230 g were anesthetized with enflurane and positioned in a stereotaxic frame. After incision of the skin and drilling 2 holes, N-methyl-d-aspartate (NMDA) solution (2 *μ*g/1 *μ*L/side) or its vehicle (saline) was bilaterally infused into the amygdala at the following stereotaxic coordinates from bregma: posterior 3.3 mm, lateral ±4.9 mm, and ventral 7.8 mm, at a flow rate of 0.5 *μ*L/min [17, 18]. Two weeks later, F3.ChAT cells (1 × 10^6^ cells/10 *μ*L/rat) or their vehicle (saline) was transplanted in rats showing loss of fear to cat odor via intracerebroventricular injection (coordinates: posterior 0.9 mm, lateral 1.4 mm, and ventral 3.6 mm from bregma). All experimental procedures were approved by the Institutional Animal Care and Use Committee of Laboratory Animal Research Center at Chungbuk National University, Korea.

### 2.3. Locomotor Activity Analysis

Locomotor activities were measured using a video tracking system (Smart v2.5; Panlab Technology, Barcelona, Spain), connected to a CCTV (Samsung, Changwon, Korea), immediately before and 2 and 4 weeks after cell transplantation [14]. Rats were placed at the center of a quiet dim chamber (60 cm × 60 cm) with fresh cat feces (10 g) along one bottom line. The movements of the rats were video-tracked for 5 min to analyze the location where the animals stayed and densitometrically recorded. In addition, the types of movement, that is, resting and slow-moving and fast-moving times, were recorded, and the ratio was analyzed.

### 2.4. ACh Analysis in Cerebrospinal Fluid (CSF)

All the rats were sacrificed at the end of locomotor testing, and CSF was collected to analyze ACh contents [1, 13]. ACh concentration in CSF was measured with the Amplex Red acetylcholine/acetylcholinesterase assay kit (Molecular Probes). In this assay, ACh is hydrolyzed by acetylcholinesterase to release choline, which is then oxidized by choline oxidase to betaine and H_2_O_2_. H_2_O_2_ interacts with Amplex Red (7-dihydroxyphenoxazine) in the presence of horseradish peroxidase to generate the highly fluorescent resorufin. The resulting fluorescence was measured in a fluorescence microplate reader using excitation in the range of 530–560 nm and emission at ~590 nm.

### 2.5. Microscopy and Immunohistochemistry in Brain Sections

The rat brains were perfusion-fixed with 4% paraformaldehyde solution and post-fixed for 48 hr, followed by cryoprotection in 30% sucrose solution for 72 hr. Coronal cryosections of 20-*μ*m thickness were prepared and processed for general staining and immunostaining. After hematoxylin-eosin or Nissl staining, the lesions in the amygdala were examined under a light microscope, and the survived cells were counted. Separately, a part of the slides were subjected to double immunostaining using antibodies specific for human mitochondria (hMito; 1 : 200, mouse monoclonal, Chemicon) and/or ChAT (1 : 200, rabbit polyclonal, Chemicon). Brain sections were incubated with primary antibodies overnight at 4°C and with secondary antibodies conjugated with Alexa Fluor 488 or Alexa Fluor 594 (1 : 1,000, Molecular Probes) for 1 hr at RT as described above.

### 2.6. Statistical Analysis

Data are presented as mean ± SEM. The statistical significance between group comparisons for behavioural data was determined by one-way analysis of variance (ANOVA), followed by post hoc Tukey's multiple comparison test. A value of *P* < 0.05 was considered to be statistically significant.

## 3. Results

**Figure 1:**
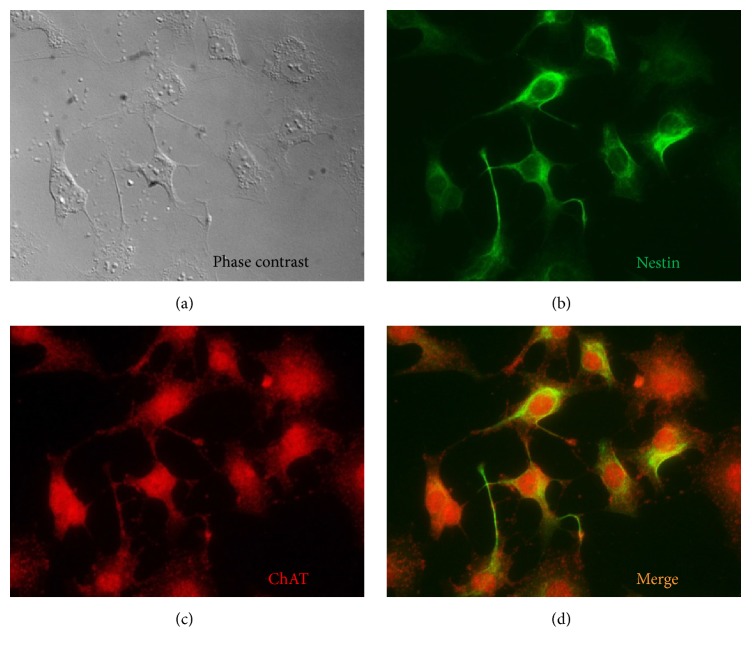
Establishment and immunophenotyping of F3.ChAT human neural stem cell line. (a) A phase contrast image of F3.ChAT cells. (b) Immunocytochemical identification of nestin protein (green) in F3.ChAT cells. (c) Immunocytochemical identification of choline acetyltransferase (ChAT) protein (red) in F3.ChAT cells. (d) Merge of nestin- (b) and ChAT-positive (c) images.

F3.ChAT cells overexpressing human ChAT gene were successfully established using a retroviral vector. In immunostaining, most of the F3.ChAT cells were double positive for human nestin (a neural stem cell marker) and ChAT (Figure 1), indicating that transfection of ChAT gene did not modify NSC features originated from parental F3 cells, excepting higher expression of ChAT gene [1, 13, 14].

**Figure 2:**
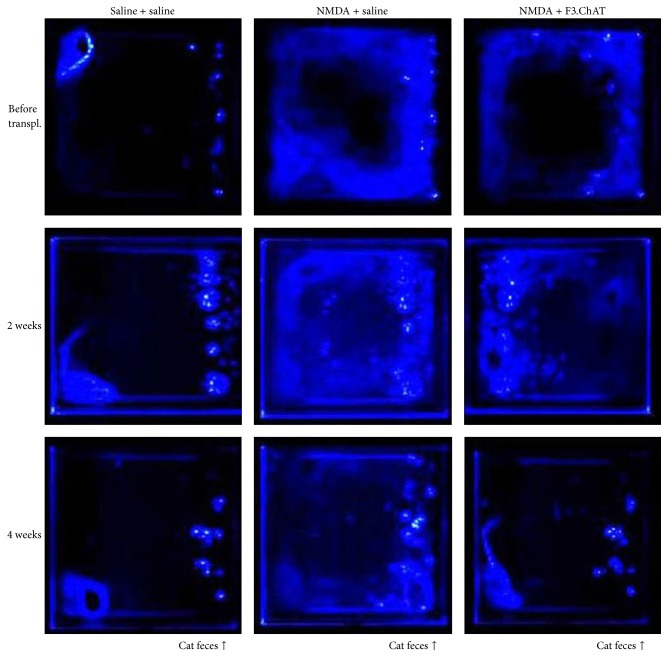
Densitometry on the movement of rats exposed to predator odor source along the right bottom line (cat feces, arrows). The blue prints were drawn by tracing motions of the rats. N-Methyl-d-aspartate (NMDA) was injected 2 weeks prior to F3.ChAT cell transplantation.

Vehicle (saline)-treated animals smelling cat odor exhibited fear freezing behaviour, staying at a corner remote from the location of cat feces (Figure 2, left panels). Such fear response lasted throughout the 6-week experimental period. However, the rats with amygdala injury by NMDA injection did not show any freezing behaviours; that is, they freely moved all around the frame, indicative of the loss of fear feeling (middle panels). By comparison, transplantation of F3.ChAT cells recovered the fear freezing behaviour from 2 weeks after transplantation, leading to a full restoration similar to normal animals (right panels).

**Figure 3:**
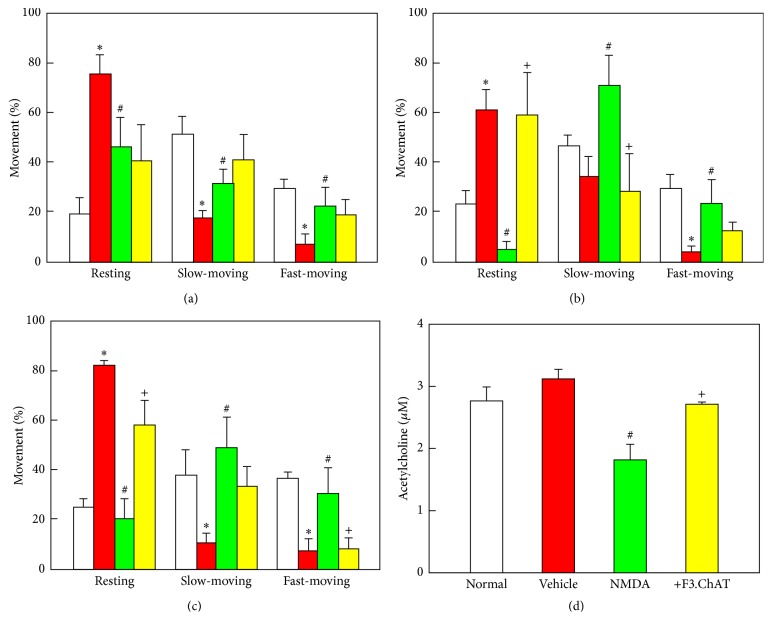
Locomotor activity of rats that encountered cat odor (a–c) and acetylcholine concentration (d). Spontaneous movements (resting and slow-moving and fast-moving times) were analyzed immediately before (a) and 2 weeks (b) and 4 weeks (c) after F3.ChAT cell transplantation following N-methyl-d-aspartate (NMDA) injection (−2 weeks), and acetylcholine was analyzed in cerebrospinal fluid 4 weeks after transplantation (d). White, normal environment (without cat odor) + vehicle (saline for NMDA and cells); red, cat odor + vehicle; green, cat odor + NMDA + vehicle; yellow, cat odor + NMDA + F3.ChAT cells. ^*∗*^Significantly different from normal control (*P* < 0.05). ^#^Significantly different from vehicle control (*P* < 0.05). ^+^Significantly different from NMDA alone (*P* < 0.05).

In locomotor activity analysis on the movement types, the rats exhibited active movements (75–81% slow- and rapid-moving times) in the normal environment without cat feces, leading to only 19–25% resting time (Figures 3(a)–3(c)). However, the resting time of animals facing cat feces odor significantly increased to 61–82% (*P* < 0.05), because of the fear freezing behaviour as seen in Figure 2. In contrast, the freezing time of NMDA-challenged rats drastically decreased, resulting in the significant increases in slow- and fast-moving times (*P* < 0.05) (Figures 3(a)–3(c)), implying that amygdala lesion causes deficit of fear feeling. Notably, transplantation with F3.ChAT cells reversed the animals' activity, markedly decreasing moving latency from 2 weeks after transplantation (*P* < 0.05).

In comparison with a slight increase in ACh concentration in CSF of animals exposed to cat odor, NMDA challenge markedly decreased the level by 40% (*P* < 0.05) (Figure 3(d)). However, such a decrease in the ACh level was significantly recovered as measured 4 weeks after transplantation of F3.ChAT stem cells (*P* < 0.05).

**Figure 4:**
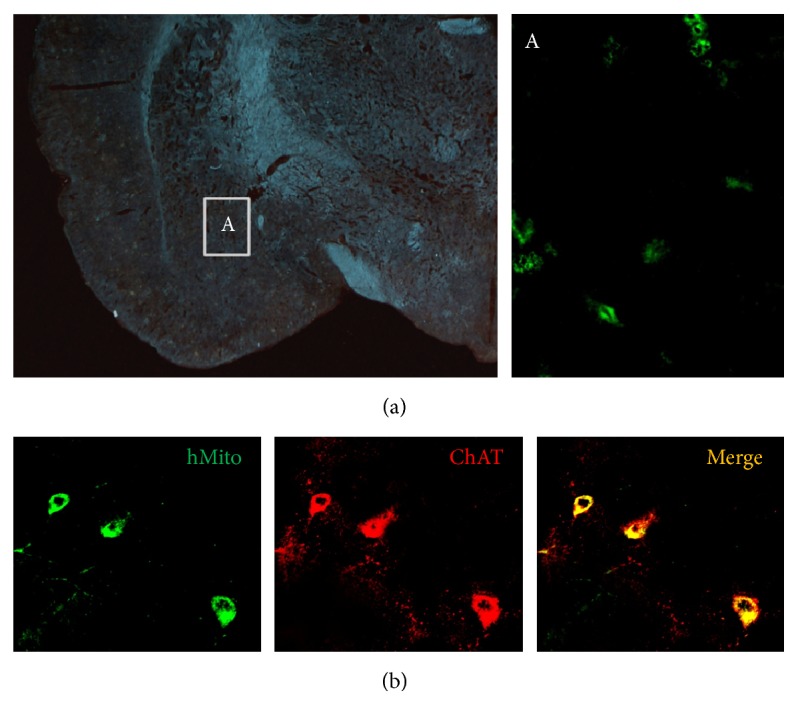
Representative images for detection of transplanted F3.ChAT cells in the amygdala (a) and for production of functional ChAT protein (b) 4 weeks after transplantation. ((a), inset A) For detection of human F3.ChAT cells, the amygdala tissue was immunostained with hMito antibody. (b) The cells were double immunostained with antibodies specific for hMito and ChAT. NMDA, N-methyl-d-aspartate; hMito, human mitochondria; ChAT, choline acetyltransferase.

After the intracerebroventricular transplantation of F3.ChAT cells (1 × 10^6^ cells/rat), transplanted human (hMito-immunoreactive) cells were detected in the amygdala [Figure 4(a) and inset (A)]. In order to confirm whether the transplanted cells express ChAT* in vivo*, a double immunostaining for hMito and ChAT was performed. The results indicate that most of the hMito-positive F3.ChAT cells produce ChAT protein, suggestive of the expression of functional gene ChAT (Figure 4(b)).

**Figure 5:**
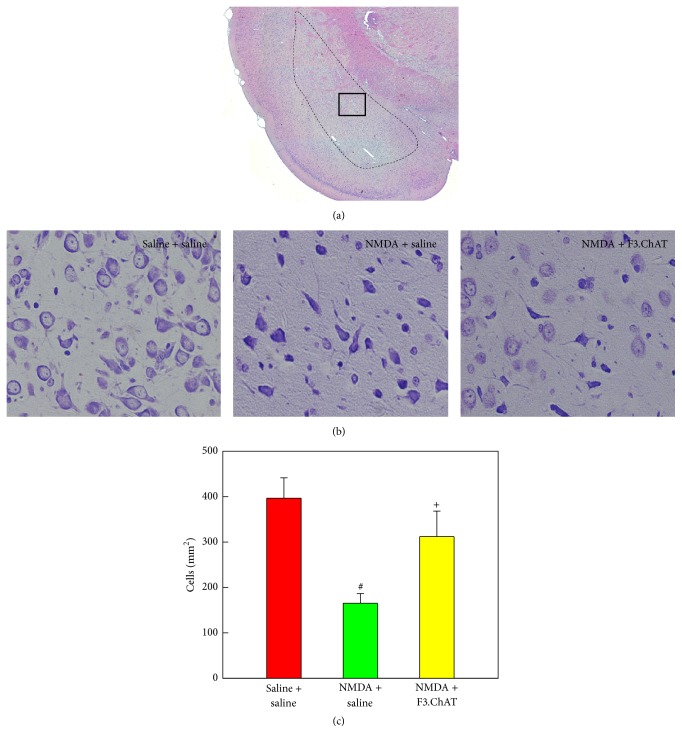
Representative hematoxylin-eosin (a) and Nissl staining (b) images of the amygdala, and the cell number in the amygdala (c) 4 weeks after transplantation. After examination on the entire amygdala (dotted line in (a)), magnified medial amygdala (inset in (a)) was photographed (b), and the number of survived neurons was counted (c). NMDA, N-methyl-d-aspartate. ^#^Significantly different from vehicle control (*P* < 0.05). ^+^Significantly different from NMDA alone (*P* < 0.05).

In hematoxylin-eosin staining on the amygdala, NMDA administration caused malacia of neuropils (Figure 5(a)). In Nissl staining, neuronal degeneration (shrinkage) was observed in the amygdala lesions, resulting in the decrease in survived neurons to 42% of normal level (*P* < 0.05) (Figures 5(b) and 5(c)). Interestingly, the NMDA-induced neurotoxicity was remarkably attenuated by treatment of F3.ChAT cells, leading to a significant increase in the neuronal survival (*P* < 0.05) (Figures 5(b) and 5(c)).

## 4. Discussion

In the present study, human NSCs overexpressing ChAT gene were successfully established, without loss of the stem cell nature (nestin-positive) after gene insertion (Figure 1). Transplantation of the F3.ChAT cells restored fear freezing behaviour of rats with NMDA-induced amygdala injury up to the level comparable to normal animals (Figures 2 and 3).

Intracerebroventricularly transplanted F3.ChAT cells survived in the brain tissue without immunosuppressant injection and migrated to the injured amygdala as observed 4 weeks after transplantation (Figure 4). Such a lesion-tropism was also confirmed in our previous studies [1, 13], in which a remarkable migration was observed at 1 week, and the cells were detected only at the lesion sites 4 weeks after transplantation. It was confirmed that most of the hMito-positive F3.ChAT cells produced ChAT protein (Figure 4), indicating that the transplanted stem cells functioned in the host brain tissue, which was further supported by the increased ACh levels in CSF (Figure 3(d)).

It is well known that direct administration of excitatory amino acids such as NMDA and kainic acid destroyed neurons in the injection sites [1, 17, 18]. In order to exclude spontaneous recovery, renewal, or reinstatement of fear extinction following injection of receptor antagonists/agonists such as antimuscarinic scopolamine and inhibitory *γ*-aminobutyric acid or extinction training [4, 7, 10, 19, 20], we injected NMDA, an excitotoxin, leading to long-term fear deficits [1, 17, 18]. Actually, the rats challenged with NMDA did not display fear freezing behaviour in 2 weeks, but freely moved around the cat feces (Figure 2). In locomotor activity, the moving times of amygdala-injured rats greatly increased compared to vehicle (saline)-treated animals (Figure 3(a)). Such phenomena lasted up to 6 weeks after NMDA injection (Figures 3(b) and 3(c)). However, F3.ChAT cell transplantation markedly recovered the fear freezing behaviour, decreasing the locomotor activity, along with the increase in ACh level (Figure 3(d)), suggestive of the key role of ACh in the retrieval of unconditioned fear memory.

Through the unconditioned (or innate) fear, animals and humans instinctively respond to threatening events without former experiences [7, 19]. Neural circuits and signaling mechanisms on the fear conditioning and recall of the conditioned fear memory have been profoundly studied [21], showing that cholinergic (nicotinic and muscarinic) ACh receptors (AChRs) and metabotropic glutamate receptor-type 5 (mGluR5) in the amygdala and hippocampus play central roles [10, 19, 20, 22–24]. In addition, protein synthesis and NMDA receptor activity-mediated dl-*α*-amino-3-hydroxy-5-methyl-4-isoxazole propionic acid (AMPA) receptor trafficking were required for memory retrieval [25]. Such results indicate that ACh and glutamate are the major neurotransmitters for fear memory similar to the habituation memory as seen in AD and aging [1, 13, 14]. Although NMDA receptor antagonists blocked acquisition of conditioned fear-potentiated startle [26, 27] and trafficking of NMDA-AMPA receptors was required for memory retrieval [25], there is no clear evidence of the roles of NMDA and AMPA receptors in the retrieval of unconditioned fear memory. In fact, it was reported that conditioned and unconditioned stimuli increased frontal cortical and hippocampal ACh release and activated basal forebrain cholinergic neurons via cortical and hippocampal projects, leading to arousal of rats [28]. In addition to the effects in AD [1, 13] and aging [14] animals, F3.ChAT cells restored the cognitive function of fear-deficit animals by increasing ACh level. Therefore, it is strongly suggested that ACh might be a key molecule for the retrieval of fear memory. To our knowledge, the present study may be the first report on the retrieval (recall) of fear without conditioned acquisition via aversive stimuli or extinction training with nonthreatening cues as in passive avoidance and water-maze trials.

It was reported that the medial amygdala plays a specific role for the retrieval of unconditioned fear [6]. In the present study, neuronal degeneration in the amygdala was remarkably attenuated by F3.ChAT cell treatment as observed 4 weeks after transplantation (Figures 5(b) and 5(c)). In our previous study, it was demonstrated that F3.ChAT cell transplantation restored microtubule-associated protein 2, a neuronal cytoskeletal protein, and especially cholinergic nerve markers in aging mice, which might be mediated by neurotrophic factors released from the stem cells [14]. Therefore, it is not excluded that neuroregeneration in the amygdala of F3.ChAT-treated rats also contributed to the restoration of ACh level and fear memory retrieval, as suggested in previous observations that administration of d-cycloserine enhanced the expression of NR2B protein, an NMDA receptor subunit, by increasing proliferation of newly born cells, and thereby facilitated the acquisition and retrieval of extinction memory [29]. If it is true, it is assumed that the amygdala may not be a storage site of fear memory but a portal for input (basolateral amygdala) and recall (medial amygdala) of the memory as hippocampus for habituation memory, since the amygdala as a reservoir (structurally modified synapses) for fear memory was destroyed already by NMDA 2 weeks before F3.ChAT cell transplantation [21].

## 5. Conclusion

Although many aspects including nervous systems, signaling molecules, and neuroregeneration related to F3.ChAT cells' action mechanism remain to be clarified, it was demonstrated that F3.ChAT cells triggered retrieval of unconditioned fear memory from amygdala-injured rats by recovering ACh concentration. In spite of the mysteries on the nature and location in the brain, the present study may provide a clue for insight into the processing and retrieval of unconditioned (instinctive or innate) fear memory.
